# Interactive structural color displays of nano-architectonic 1-dimensional block copolymer photonic crystals: FOCUS ISSUE REVIEW

**DOI:** 10.1080/14686996.2022.2156256

**Published:** 2023-01-04

**Authors:** Tae Hyun Park, Seunggun Yu, Jeongok Park, Cheolmin Park

**Affiliations:** a Laboratory of Organic Electronics, Department of Science and Technology, Linköping University, Norrköping, Sweden; b Insulation Materials Research Center, Korea Electrotechnology Research Institute, Changwon, Republic of Korea; c College of Nursing, Mo-Im Kim Nursing Research Institute, Yonsei University, Seoul, Republic of Korea; d Department of Materials Science and Engineering, Yonsei University, Seoul, Republic of Korea

**Keywords:** Self-assembly, block copolymer, structural color, photonic crystal, programmable structural color, stimuli-interactive structural color

## Abstract

For changing environmental circumstances, interactive structural color (SC) observation is a promising strategy to store and express external information. SCs based on self-assembled block copolymer (BCP) photonic crystals have been a research focus due to their facile and diverse nanostructures relying on the volume ratio of blocks. Their unique nano-architectonics can reflect incident light due to constructive interference of the two different dielectric constituents. Their excellent ability to change nano-architectonics in response to external stimuli (i.e. humidity, temperature, pH, and mechanical force) allows for a programmable and stimuli-interactive BCP SC display. In this review, recent advances in programmable and stimuli-interactive SC displays with the 1-dimensional self-assembled BCP nano-architectonics are comprehensively discussed. First, this review focuses on the development of programmable BCP SCs that can store various information. Second, stimuli-interactive BCP SCs capable of responding reversibly to external stimuli are also addressed. Particularly, reversible BCP SC changes are suitable for rewritable displays and emerging human-interactive BCP SC displays that detect various human information through changes in electric signals with the simultaneous alteration of the BCP SCs. Based on previously reported literature, the current challenges in this research field are further discussed, and the perspective for future development is presented in terms of material, nano-architectonics, and process.

## GRAPHICAL ABSTRACT

**Figure:**
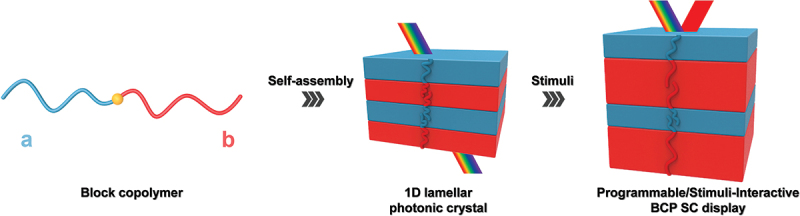

## 1. Introduction

Structugral colors (SCs) of photonic crystals (PCs) resulting from constructive interference of light with periodic nano-architectonics of two different dielectric constituents are of great interest due to their potential use in low-power reflective mode displays, information storage, and sensors [1–5]. PCs based on self-assembled block copolymers (BCPs) are additionally beneficial as their periodicities and dielectric constants are readily altered by various external stimuli, such as electric field (E-field) [6–11], humidity [12,13], temperature [14], and mechanical forces [15]. This allows for the facile stimuli-interactive change, making these PCs potentially suitable for various emerging actively programmable and stimuli-interactive display elements.

Due to the relatively small period of normal molecular weight BCPs typically having domain dimensions of tens of nanometers, the maximum reflection in a BCP PC frequently occurs at UV or higher photon energies; this makes the SC of such BCPs rarely realized in the visible range [16]. Visible-color BCP PCs with ultra-high molecular weights were demonstrated, but they exhibited an inherently slow response to external stimuli due to extremely slow molecular kinetics [17,18]. Although BCPs with designed molecular architectures, particularly the bottle-brushed type molecular architecture, were developed with visible range SCs [19–24], the synthesis of BCPs was not trivial and required a careful control of multi-step reactions. A solvent capable of swelling the domains is often employed to increase the domain spacing of a BCP and to impart SC in the visible range [25–29]. The development of a quaternization process that allowed the facilitation of solvent infusion into one of the alternating BCP lamellae with quaternized amine moieties in the chains [8,25,28] was an important achievement. The fast preferential swelling of the quaternized domains resulted in the SCs in the full-visible range of the BCP upon exposure to the solvent. Since this instrumental work, numerous studies to control the degree of swelling of the domains have been accomplished to manipulate the visible SCs of a BCP not only in liquidous states [26–44] but also in thin solid films [12,13,45–50]. These methods included the addition of salts [31–37], the control of pH [38–41], the control of cross-linking of the swollen domains [42–44], and the manipulation of temperature [14,47], humidity [12,13], pressure [15,48], and external E-field [6–11,49,50].

The facile alteration of BCP SCs using various stimuli facilitates the development of displays, allowing the direct visualization of the information programmed and stored in non-volatile manners; this makes them potentially suitable for various emerging smart technologies, such as pattern-based identification, sensing, and encryption [28,42–44]. The diverse information of symbols, characters, and images is reported using an external stimulus to vary the SC in BCP PCs when the SC change is localized and 2-dimensionally spatially addressed. Considering that most BCPs employed for PCs are solution-processed in ambient conditions, solution-based printing technologies, such as ink-jet and micro-contact printing, are extensively employed for storing information. Recent research shows that stimuli-interactive BCP SCs are potentially suitable for emerging human-interactive displays. Various mechanical and physiological human information is electrically detected and simultaneously visualized with BCP SCs in the full-visible range; these include body temperature [47], humidity [13], tactile touch [48], and finger motions [12].

**Figure 1.**
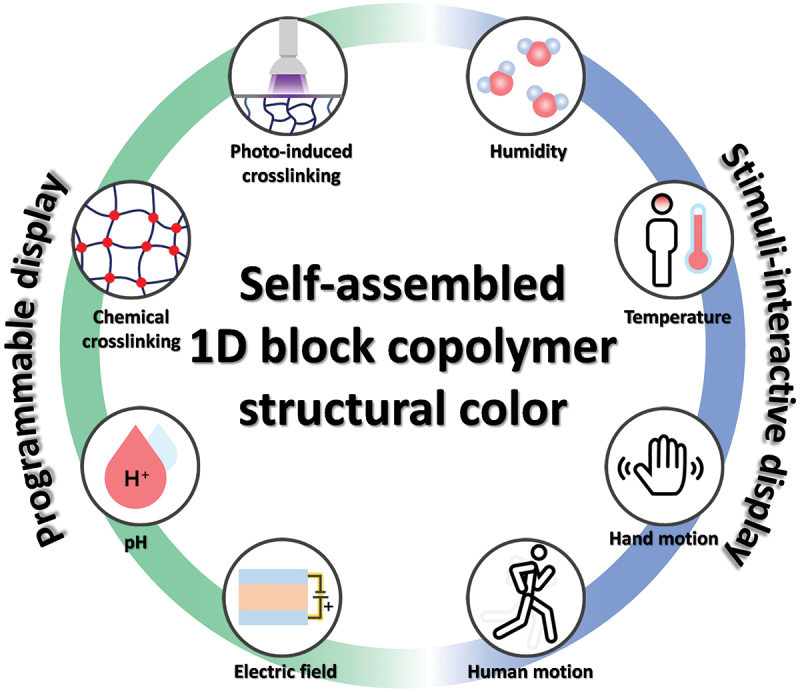
Schematic overview of programmable and stimuli-interactive structural color displays of self-assembled 1D block copolymer nano-architectonics.

Herein, an overview of the recent achievements in SC displays based on 1-dimensional (1D) self-assembled BCP PCs is comprehensively discussed. The BCP SC displays are categorized into two groups: (1) actively programmable SC displays and (2) stimuli-interactive SC displays (Figure 1). The review initially discusses the development of BCP SC displays where various information is written and stored in non-volatile manners by employing diverse stimuli-adaptive SCs of BCPs. The following section of the review deals with the development of stimuli-interactive BCP SC displays; these are suitable for re-writable BCP SC displays and the emerging human-interactive BCP SC sensors in which human information is electrically detected via simultaneous alteration of the BCP SCs with stimuli. Critical materials and process aspects, such as the design of high reflectance BCP PCs, understanding and control of the structural defects in BCP PCs, various material/nano-architectonics, and development of micropatterned BCP PCs, are emphasized to further improve the display performance for the next-generation BCP SC displays.

## 2. Actively programmable BCP displays

### 2.1. Color programmable BCP display

Self-assembled 1D BCP PCs with various lamellae periodicities were prepared by blending two poly(styrene-*b*-2-vinylpyridine) (PS-*b*-P2VP) BCPs with different molecular weights of 80 kg mol^−1^ (PS-*b*-P2VP80) and 230 kg mol^−1^ (PS-*b*-P2VP230) [28]. The lamellae periodicities of the mixed BCPs were varied linearly from 46 to 91 nm by controlling the mixing ratios of the two BCPs. The initial periodicity of PS-*b*-P2VP80 at approximately 46 nm was gradually increased by the addition of PS-*b*-P2VP230. For a neat PS-*b*-P2VP230 solution, a periodicity of approximately 93 nm was observed. The SC of a binary blend BCP PC film was developed when the BCP PC film was immersed in ethanol; this preferentially swelled the quaternized-P2VP (QP2VP) domains of the binary BCP film. The SC of a neat PS-*b*-QP2VP80 film in ethanol was observed in the UV region at a maximum reflection of 380 nm. The SC was tuned in the visible range by adding PS-*b*-QP2VP230 to PS-*b*-QP2VP80. The wavelength at the maximum reflection increased linearly from 388 to 762 nm with the increase in the PS-*b*-QP2VP230 ratio in the binary BCP PCs.

**Figure 2.**
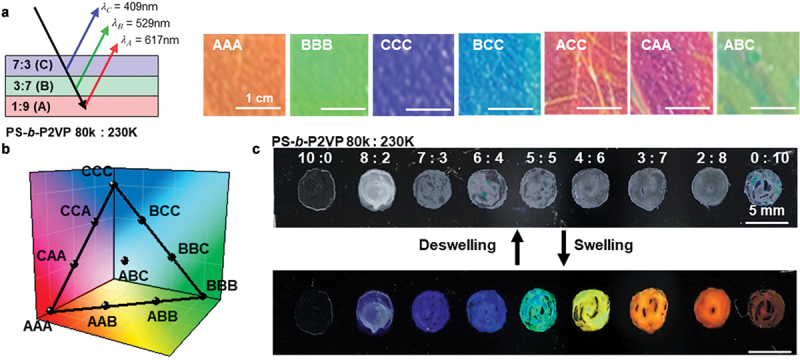
(a) (left) Schematic of SC encryption by combining three binary BCP PCs of PS-*b*-QP2VP80:230 (1:9) (a), (3:7) (b), and (7:3) (c). (right) Photographs of seven SCs of the stacked binary BCP PC films when immersed in ethanol. (b) 3D color coordinates of ten combinations of the three BCP PC stacking. (c) Optical microscopy (OM) images of a hand-drawn BCP SC of binary PC palette with various mixing ratios with dot shape before swelling/deswelling. Reproduced with permission [28]. Copyright 2022, Wiley-VCH.

For the binary blends of BCP PCs with SCs in the full-visible range, a binary BCP PC palette was used for various solution-based thin-film processes, such as spray, bar, and spin coating; this facilitated the development of the PC films on target surfaces with various geometries and topologies. The blending solution was successfully spray-coated onto the topological surface of a mussel shell, resulting in a red SC when immersed in ethanol. The binary BCP PC films were potentially suitable for reflection mode-pattern encryption. By stacking binary BCP-blend films with a mechanical transfer process, the SCs of the corresponding films were readily mixed, resulting in combined SCs with a controlled reflection intensity and wavelength. For the demonstration of pattern encryption based on three state SCs, the three BCP PC films chosen were neat PS-*b*-QP2VP80:230 (1:9) (A), PS-*b*-QP2VP80:230 (3:7) (B), and PS-*b*-QP2VP80:230 (7:3) (C) with red, green, and blue SCs, respectively, using the preferential swelling of QP2VP domains in ethanol (Figure 2(a)). Various combinations of the three PC layers (AAA, BBB, CCC, ABB, AAB, BCC, BBC, CCA, CAA, and ABC) were examined, and the results showed that the reflection intensity and position were readily controlled by a suitable selection of the BCP PCs. The encrypted SCs resulting from the combination of the three PC layers were instantly visualized and produced various mixed SCs in the visible range. A total of 10 combinations of different mixed SCs based on the three-layered BCP PCs were plotted on a triangular plane in 3D color coordinates (Figure 2(b)). The results showed that various SC coding was obtained from the numerous combinations of the encrypted BCP PC layers. The binary BCP PC palettes were also suitable for the application in 2-dimensional (2D) full-color pattern encryption drawn using commercial brushes (Figure 2(c)). Using a fountain pen consisting of a paintbrush and PC palette, a full-color SC painting was developed using transparent PC ink to facilitate a programmable low-power 2D pattern encryption.

### 2.2. Photo-programmable BCP display

UV irradiation at 365 nm gradually induced the photo-crosslinking of QP2VP layers with increasing exposure time; changing the SC from red to green to blue and then to transparent for a PS-*b*-QP2VP BCP PC [42]. The anion species that pair with the pyridinium groups of the QP2VP layer modulated the photosensitivity of the BCP SC. The SC of BCP PC exchanged by acetate and phenoxide anions exhibited dramatic transition curves and a large blue shift, whereas the SC of BCP PC exchanged by bromide or iodide anions was rarely changed in its SC. Since most photo-crosslinking is associated with the radical formation on the backbone or the side-chain polymers, the counter-anions affected the efficiency of radical formation or radical quenching. This anion-dependent photo-crosslinking enabled the development of a multi-color BCP SC display. A BCP PC modified with highly photosensitive phenoxide ions exhibited an initial red SC before UV irradiation, and then became green after UV irradiation. The second anion exchange from phenoxide to iodide was conducted, and the SC still remained green after further UV irradiation. Anion exchanges from iodide to phenoxide re-activated the fixated BCP PC. A 2D multicolor check-pattern encryption was fabricated by using double photolithography with a 1D periodic line-pattern mask by rotating the shadow mask at 90°. The resolution of the line pattern was approximately 8 μm.

**Figure 3.**
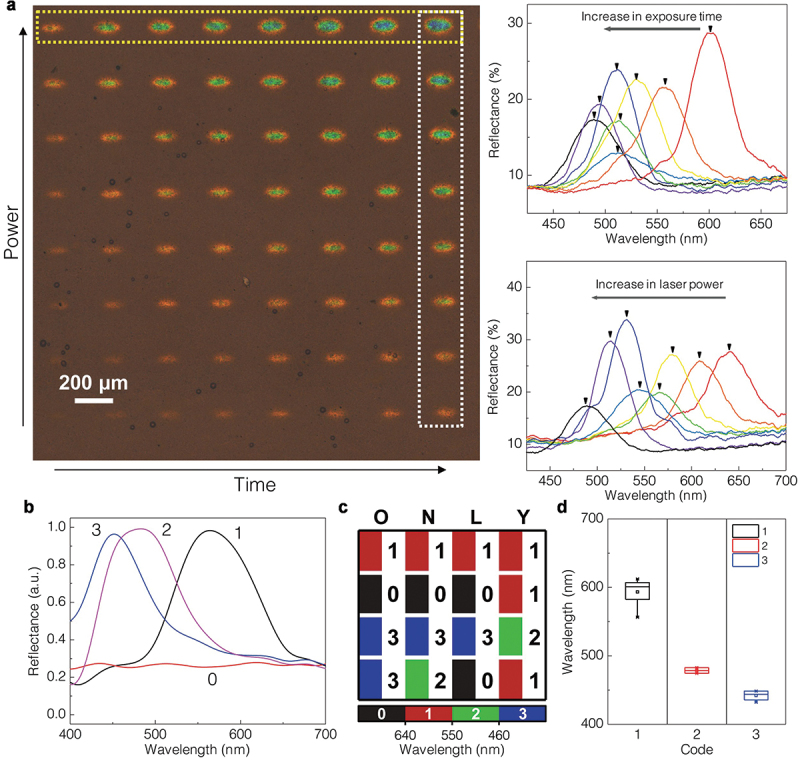
(a) (left) OM image of a photo-programmable BCP SC display by a laser after immersion in ethanol and (right) vis spectra of BCP SCs as a function of exposure time and laser power. (b) Vis spectra of BCP PCs as a function of laser power for quaternary BCP SC patterns. (c) the letter ‘ONLY’ in ASCII code converted using a quaternary SC code system; four digits 1033, 1032, 1030, and 1121 represent O, N, L, and Y, respectively. Transparent SC (0 in quaternary code), red SC (1 in quaternary code), green SC (2 in quaternary code), and blue SC (3 in quaternary code). (d) Reflected wavelengths corresponding to quaternary BCP SC of (c). Reproduced with permission [43]. Copyright 2019, Wiley-VCH.

Eoh et al. developed a write once read many (WORM) BCP SC display in which non-volatile information written by laser illumination on a BCP PC was displayed in SCs in the full-visible range [43]. The WORM SC display was based on the photo-thermally induced cross-linking of QP2VP domains. Heat was produced in a photothermal layer of a poly(3,4-ethylenedioxythiophene) doped with tosylate (PP-PEDOT) using laser illumination and transferred to a BCP PC placed on the PP-PEDOT layer. A thermally active cross-linking agent of dibromobutane (DBB) triggered the cross-linking of the QP2VP domains. An SC of the cross-linked BCP PC was blue-shifted with an increase in the laser exposure time. By controlling the crosslinking of the QP2VP domains using both the initial concentration of DBB in the solution and laser exposure, various SCs of the BCP PCs were developed in the visible region. This study showed that the irreversible cross-linking of the QP2VP domains was attributed to the thermal radical generation of the QP2VP chains. Photothermal conversion using a PP-PEDOT layer was dependent on laser power and exposure time. The photo-energy of a focused laser beam was converted to thermal energy, which was proportional to the laser power; this allowed the precise programming of the cross-linking positions of the QP2VP layers (Figure 3(a)). A PS-*b*-QP2VP film with a red SC was prepared on the PP-PEDOT layer for a WORM-type memory-bit operation. By adjusting the exposure time from 1 to 8 s and the laser power in the range of 3.4–4.9 μW μm^−2^, a total of 8 × 8 matrix memory bits were recorded; this was suitable for high-density programmable 2D information storage.

To demonstrate a multi-color, multilevel information storage display using a photo-programmable BCP PC, wherein the localized SC was controlled by both the laser power and exposure time, the existing ASCII codes were replaced with four representative SCs (0: near-infrared (NIR), 1: red, 2: green, and 3: blue) that were selected from the BCP PC (Figure 3(b)). Using various combinations of these four SCs corresponding to the ASCII codes, the word ‘ONLY’ was spelled (Figure 3(c)). Distinguishable wavelength windows with narrow spot-to-spot variations were obtained with the SCs locally written by photothermal conversion of the PP-PEDOT layer with the laser (Figure 3(d)). The reflectance spectra indicated that the combined spots denoting the letters in ‘ONLY’ were successfully stored and displayed using the localized SCs with different wavelengths of maximum reflection.

### 2.3. Chemically programmable BCP display

Kang et al. developed an inkjet-printable BCP SC display based on chemical crosslinking and de-crosslinking of the QP2VP domains with ammonium persulfate (APS) ink and hydrogen bromide (HBr), respectively [44]. This work showed that APS dissolved in water/ethanol cosolvent efficiently cross-linked the QP2VP domains through the ion exchange between QP2VP+-Br- and (NH_4_+)SO_3_-OOSO_3_-(NH_4_+). The as-prepared BCP SC film exhibited a wavelength at the maximum reflection of 700 nm owing to the exposure to water/ethanol. The degree of swelling of the QP2VP domains was decreased with the amount of APS in water/ethanol solution; this resulted in a blue-shift of the wavelength at the maximum reflection of the BCP SCs with APS. By optimizing APS in the solution, the SC of a BCP PC was controlled over a broad wavelength range from NIR to the UV regions.

**Figure 4.**
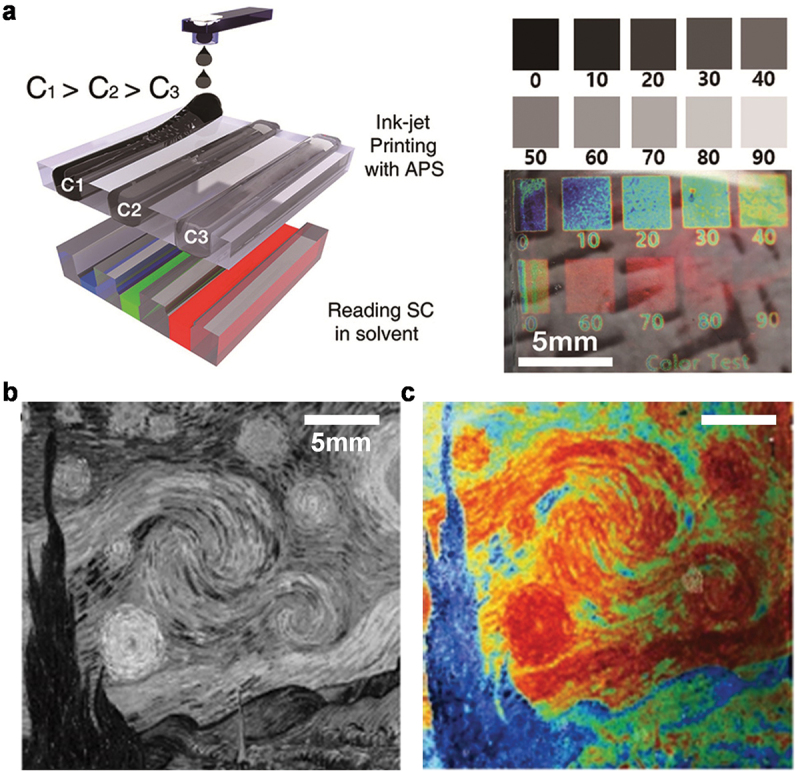
(a) (left) Schematic of a printable APS ink in the cartridge of a modified inkjet printer with different APS concentrations C_1_, C_2_, and C_3_. the APS concentration increases in the order of C_1_, C_2_, and C_3_ (C_1_ < C_2_ < C_3_). (right) a black and white contrast for regulating APS concentration in a specific area. Photographs of the inkjet-printed BCP SCs at corresponding contrast when immersed in ethanol. A low contrast number representing a high APS concentration. (b) Black and white contrast image converted by computer software. (c) Resulting BCP SC image printed by APS ink when immersed in ethanol. Reproduced with permission [44]. Copyright 2017, Wiley-VCH.

A modified inkjet printer was used to directly jet the APS ink in the cartridge onto a BCP PC film (Figure 4(a)). Since the SC of the BCP PC film was blue-shifted with APS when the BCP PC film was immersed in the solution, the inkjet printer programmed the quantity and position of APS on a BCP PC film. For demonstration, an original colorful information image was initially converted into a black and white contrast one (Figure 4(b)). The ink-jet printing was then performed on the BCP PC film with a solution of APS in water/ethanol to develop an ink-jetted image of the black and white one. In this process, the position and density of the cross-linking of the QP2VP domains in the BCP PC film were controlled. When the APS-programmed BCP PC film was immersed in ethanol, the original color image was successfully developed in the BCP SC display (Figure 4(c)). To de-crosslink the APS-treated QP2VP domains, the BCP PC film was treated with HBr. The SC of a BCP PC programmed with APS was red-shifted with the HBr treatment due to the enhanced swelling of the QP2VP domains after the de-crosslinking. The work demonstrated that the reversible crosslinking and de-crosslinking with APS and HBr, respectively, occurred for more than fifty cycles. An SC image in the full-visible range developed on a BCP PC film with an APS ink was completely erased upon HBr treatment, and a new color image was readily developed with another APS ink-jet programming.

### 2.4. PH programmable BCP display

**Figure 5.**
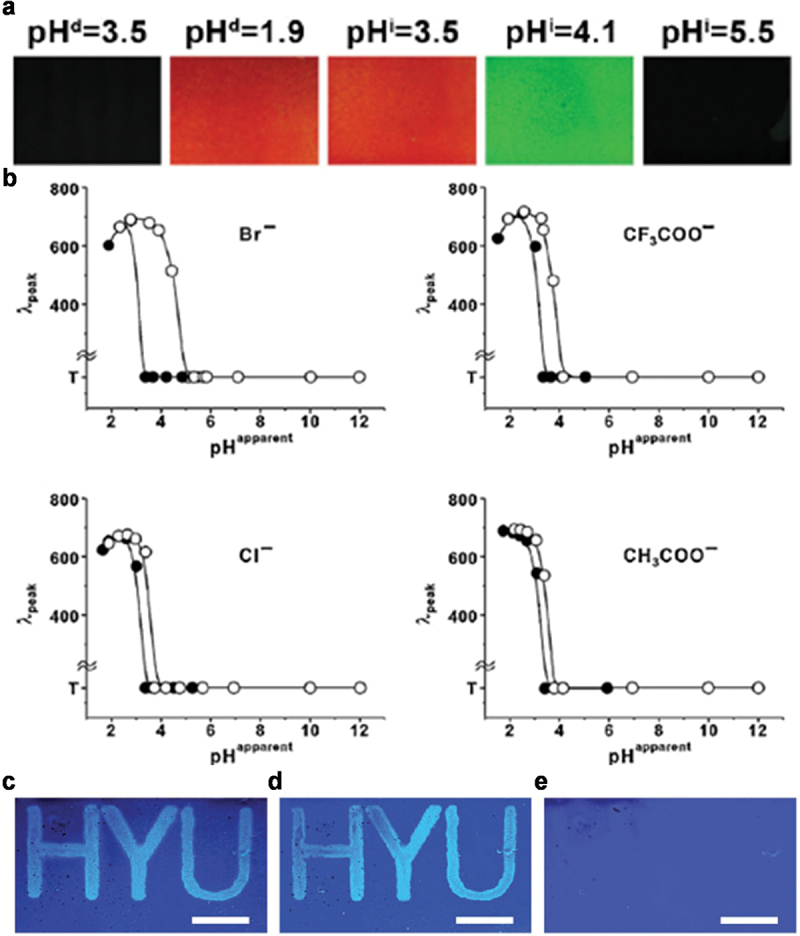
(a) Photographs of BCP PC during the sequential pH^i^/pH^d^ cycle. (b) SC hysteresis loops of BCP PC as a function of pH in HBr, CF_3_COOH, HCl, and CH_3_COOH aqueous solutions. Filled circles (●) and open circles (○) represent pH^d^ and pH^i^ cycles, respectively. The photographs of BCP SC treated with acidic ink (0.1 M HBr), followed by immersion in 0.03 M NaBr solution for (c) 12 h and (d) 96 h. (e) Photograph of erased BCP SC by washing with 0.1 M NaOH solution. Reproduced with permission [38]. Copyright 2010, Wiley-VCH.

When the pH was decreased to below the pK_a_ of pyridine, the pyridine groups of P2VP blocks were protonated; this made the P2VP layers swollen due to electrostatic repulsion between the protonated P2VP layers [38]. When the pH was increased to above the pK_a_ of P2VP, the deswelling of P2VP layers occurred due to deprotonation. BCP PCs were immersed in various aqueous solutions to decrease the pH (CH_3_COOH, HCl, HBr, HI, CF_3_COOH, HClO_4_, and HNO_3_) and increase the pH (NaOH). The wavelengths at the maximum reflectance were recorded sequentially with a decrease in pH from 6.5 to 1.5 (referred to pH^d^) and an increase in pH from 1.5 to 12 (referred to pH^i^); d and i represent the decreasing and increasing pH cycles, respectively. The reflected wavelengths recorded at the same pH during the increasing and decreasing pH cycles were not identical (Figure 5(a)). At a pH of 3.5, the BCP SC was transparent during the decreasing pH cycle and red during the increasing pH cycle. A plot of the reflected wavelength of BCP SC as a function of pH produced nonlinear SC hysteresis loops for the various acids (Figure 5(b)). The SC hysteresis loops during the increasing pH cycle were shifted to higher pH than the SC hysteresis loops during the decreasing pH cycle; the extent of this shift depended on the particular acid. The magnitude of the residual pH required to revert the SC hysteresis in the increasing pH cycle to that measured during the decreasing pH cycle is defined as the pH coercivity (pH_c_); this was dependent on the hydration-free energy correlated with the nature of the counter anions. pH_c_ increased in the following order: CH_3_COOH < HCl < HClO_4_ < CF_3_COOH < HI < HNO_3_ < HBr. The pH_c_ value of HBr was the highest among the acids and resulted in the largest SC hysteresis loop.

A BCP PC immersed in HBr with a large SC hysteresis loop served as a pH programmable BCP SC display; the information was repetitively written and erased at least 15 times, and the information written by HBr was retained for at least 96 h (Figure 5(c,e)). Initially, an acid ink (0.1 M HBr) was spray-coated for encrypting the letters ‘HYU’ on a dry BCP PC. After completely drying the BCP PC, it was immersed in 0.03 M NaBr ethanoic solution at a pH of 8. When the BCP PC film was soaked in the NaBr solution, it displayed a cyan SC in contrast to the blue SC background (Figure 5(c)). Due to the large SC hysteresis with HBr, the letters were maintained without a significant change for 96 hours (Figure 5(d)). This SC hysteresis was completely erased and reset by increasing the pH to above 12 after washing with 0.1 M NaOH solution (Figure 5(e)). The pH programmable SC was further tuned by adjusting the copolymer molecular weight.

### 2.5. Electric field-programmable BCP display

**Figure 6.**
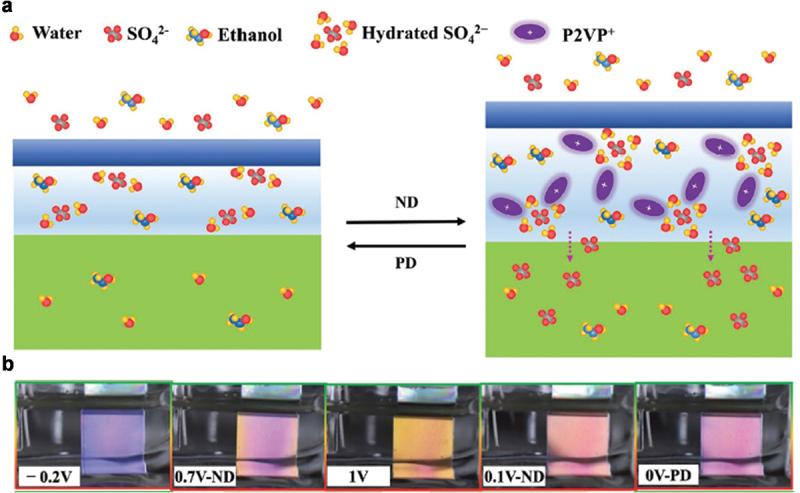
(a) Schematic of the working mechanism of SC changes during a negative direction (ND) and positive direction (PD) scan. During the ND scan, P2VP blocks were protonated (P2VP+) and attracted hydrated SO_4_^2-^. Conversely, the P2VP+ blocks were deprotonated during the PD scan and expelled hydrated SO_4_^2-^. (b) Photographs of SCs of BCP PC/PANI/ITO device at different potentials during CV cycle in the electrolyte of H_2_SO_4_-C_2_H_5_OH-H_2_O. Reproduced with permission [11]. Copyright 2021, Wiley-VCH.

The swelling and deswelling of a BCP PC were also controlled by an E-field-dependent ion diffusion; this gave rise to the development of E-field programmable BCP PC SC displays. Recently, Zhang et al. developed a BCP SC display where a BCP PC was placed on the polyaniline (PANI)/indium tin oxide (ITO) layer in the H_2_SO_4_-C_2_H_5_OH-H_2_O aqueous electrolyte [11]. The BCP SC display possessed an electrochromic and SC synergistic effect by combining an electrochromic PANI layer and a BCP PC. The pyridine group in the P2VP layers was protonated under the negative direction (ND) of the E-field and resulted in the positively charged P2VP+ (Figure 6(a)). The P2VP+ layers attracted the hydrated SO_4_^2-^ anions from the electrolyte and water molecules associated with the SO_4_^2-^ anions; this made the P2VP+ layers swollen. Conversely, the P2VP+ layers recovered their original thickness with the positive direction (PD) of the E-field because the hydrated SO_4_^2-^ anions were electrically diffused out. Without illumination, the display exhibited color changes of green, blue, and bluish violet at a specific applied voltage due only to the electrochromic response of PANI under an E-field. When the display was exposed to light, the E-field programmable SC of the BCP PC appeared with the electrochromic color from PANI. Upon ND/PD E-field cycle, the display exhibited various vivid color changes from slate blue to sandy brown to light pink to antique pink (Figure 6(b)).

Most E-field programmable BCP SC displays were operated in a liquid medium to facilitate the ion diffusion of swelling agents into a target BCP domain. The liquid-cell BCP SC displays required complex-sealing procedures for preventing the leakage and evaporation of the solvent. Park et al. demonstrated an E-field programmable soft-solid BCP SC display with a QP2VP swelling agent of Li+TFSI^−^, a non-volatile ionic liquid (IL) [49]. The display consisted of a soft ionic gel (IG) of Li+TFSI^−^ and poly(vinylidene fluoride-trifluoroethylene-chlorofluoroethylene) (PVDF-TrFE-CFE) spin-coated on a BCP PC. Depending on the concentration of Li+TFSI^−^ ions in the IG layer, BCP SC was obtained in the full-visible range. The BCP SC display was mechanically flexible and easily bent when placed on a flexible substrate. It was durable even after 1000 bending cycles with a bending radius of 3 mm. A simulation study confirmed that the water molecules were associated with the hygroscopic Li+TFSI^−^ ions, forming a hydration shell with a total coordination number of approximately 13.

**Figure 7.**
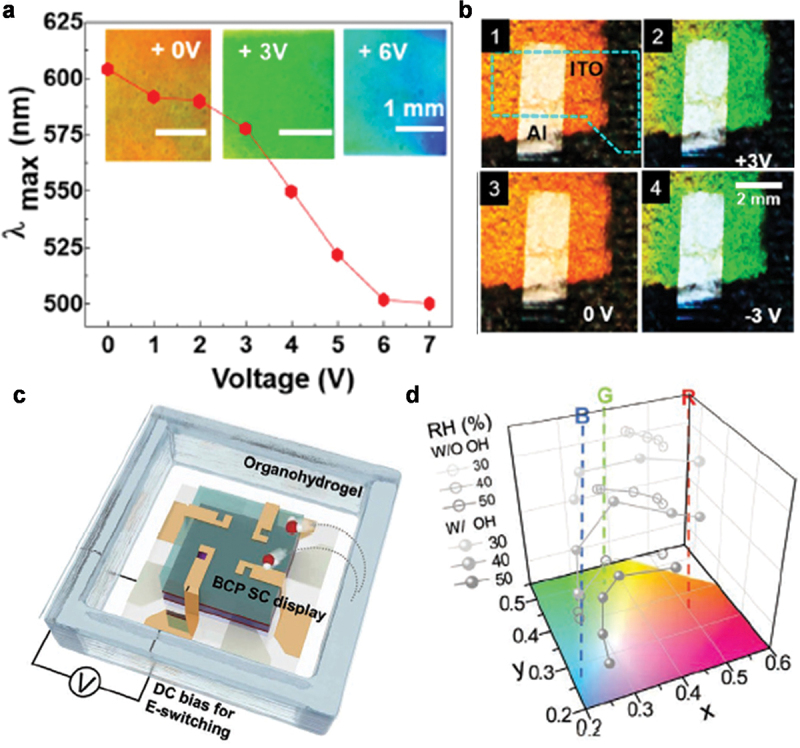
(a) Plot of wavelength at the maximum reflectance and the photographs (inset) as a function of applied voltage. (b) Photographs of E-field programmable BCP SC display exhibiting orange (at 0 V), green (at +3 V), orange (at 0 V), and green (at −3 V) during continuous voltage sweep. Reproduced with permission [49]. Copyright 2014, American chemical society. (c) Schematic of E-field programmable BCP SC display where an organohydrogel was placed near a BCP SC of PS-*b*-QP2VP/PVDF-TrFE-CFE:Li+TFSI^−^. (d) CIE coordinates of the BCP SC display as a function of humidity conditions from 30 to 50% under +2.5 V. Reproduced with permission [50]. Copyright 2022, Wiley-VCH.

For the development of an E-field programmable BCP SC display, a capacitive-type architecture was designed where the BCP PC was sandwiched between two bottom and top IG layers. When E-field was applied to the display, the SC of the display was blue-shifted due to the deswelling of QP2VP domains; this arose from the extraction of the water associated with Li+ ions from the BCP PC. The initially orange-red BCP turned green when a voltage of 3 V was applied to the top electrode, and it turned blue at an applied voltage of 6 V (Figure 7(a,b)). Without the E-field bias, the water associated with Li+ ions was moved back to the QP2VP domains, causing their swelling. The E-field programmable red-shift in the SC of the BCP PC display was observed.

The E-field-switching performance of the soft-solid BCP PC SC display was humidity-dependent due to the water associated with Li+ ions in the BCP films [50]. For example, the initial red BCP SC turned blue with increasing humidity, exhibiting maximum reflections at wavelengths of 451 nm at 90% relative humidity (RH) and 665 nm at 30% RH. High humidity induced the diffusion of Li+ ions out the QP2VP layers due to the high solubility of Li+ ions in the IG; this resulted in the blue-shift of SC. Additionally, the diffusion of Li+ ions under an E-field was significantly affected by the humidity. Due to the complicated effect of humidity on the E-field-switching performance of a BCP SC display, the E-field programmable BCP SC display was properly operated in a narrow humidity range of approximately 50% RH. To broaden the operational humidity range, an organohydrogel (OH) was employed in a BCP SC display as a permanent self-humidity source to provide an optimal humidity environment (50% RH) to the display over a long time (Figure 7(c)). An E-field programmable BCP display with a geometry-optimized OH exhibited a stable E-field switching in SCs in the full-visible range even under low RH conditions (Figure 7(d)). Moreover, a display with micropatterned BCP PC film showed a rapid response time of approximately 15 s at a low voltage due to the effective lateral diffusion of the hydrated Li+ ions through the topological patterns.

## 3. Stimuli-interactive BCP displays

### 3.1. Humidity-interactive BCP display

Kang et al. demonstrated a humidity-interactive BCP SC display by incorporating hygroscopic Li+TFSI^−^ into interpenetrated hydrogel networks (IHN) in BCP PC nanostructures [12]. Initially, oligomers were selectively infused into QP2VP and cured by UV light. Depending on the domain size of the oligomer-infused QP2VP, the IHN-BCP SC in the visible region was achieved upon UV exposure. Upon the addition of 1-ethyl-3-methylimidazolium bis-(trifluoromethylsulfonyl)-imide (EMIM+TFSI) to a given IHN-BCP PC with its initial SC, IHN-QP2VP layers were further swollen; this caused the first reflection in the BCP PC in the NIR region. The color mixing of the high-order PC reflections that occurred in the visible region led to an unprecedented SC in the visible range. When a hygroscopic Li+TFSI^−^ IL was infused into the IHN-QP2VP layer, a humidity-interactive BCP SC display was developed. When the Li+TFSI^−^ ions diffused into the QP2VP layers, the Li+TFSI^−^doped IHN BCP readily absorbed and coordinated with water molecules. The absorbed water molecules by hygroscopic Li+TFSI^−^ ions expanded the IHN-QP2VP domain size, rendering the red-shifted SC of the BCP PC with humidity; this led to humidity-interactive BCP SC displays.

**Figure 8.**
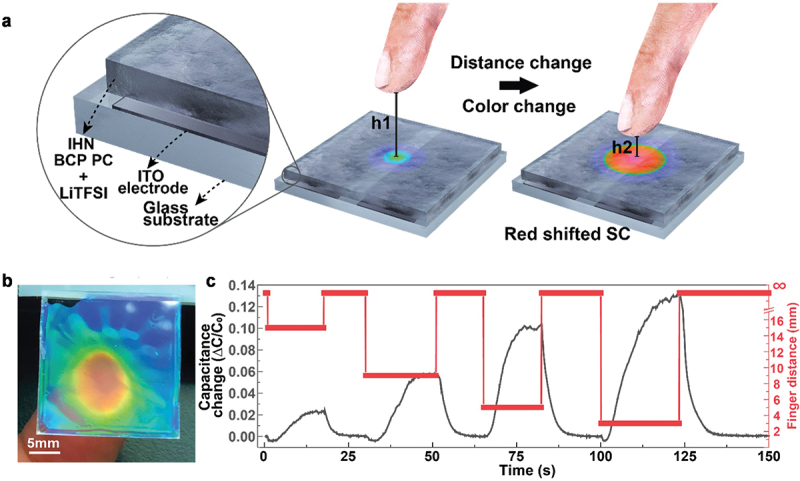
(a) Schematic of a humidity-interactive BCP SC on parallel ITO electrodes. Blue SC at height 1 (h_1_) and red SC at height 2 (h_2_) are indicated. (h_1_ > h_2_). (b) Photograph displaying humidity-interactive BCP SC change as a finger approaches the surface. (c) Capacitance change in humidity-interactive BCP SC as a function of finger-to-SC displacement from 15, 9, 5, and 3 mm. Reproduced with permission [12]. Copyright 2020, American association for the advancement of science.

The Li+TFSI^−^-doped IHN BCP was transferred onto two in-plane ITO electrodes where the capacitance change of the BCP SC was monitored with water absorption (Figure 8(a)). As an excellent source of humidity, a human finger has a natural humidity of approximately 90%. When the distance between the surface of a Li+TFSI^−^-doped IHN BCP SC and the finger varied from 1 to 15 mm, a decrease from approximately 70% to 40% in RH was observed. As the finger approached the surface, the capacitance increased because of high water uptake, which caused a red-shift in the BCP SC from blue to orange (Figure 8(b)). The capacitance change showed that each 3D location of the finger along the z-axis was distinguished repetitively (Figure 8(c)). For 3D recognition of the finger movement on the x-, y-, and z-axes, a pixelated array of humidity-interactive BCP SC on the pairs of ITO parallel electrodes was developed on a glass substrate. When the finger was located at the bottom left area of the humidity-interactive BCP SC display, the 3D location of the finger was readily reconstructed with the capacitance change. The reversible SC change was highly sustainable even after repetitive cycles. Therefore, 3D-finger movements were detected in the capacitance change and simultaneously visualized in the BCP SC variation.

### 3.2. Temperature-interactive BCP display

As discussed in 3.1, the absorption and desorption of water molecules in a BCP PC play an important role in controlling the stimuli-interactive SCs. In this regard, temperature-controlled water evaporation from Li+ ions is an excellent strategy for developing a temperature-interactive BCP SC display. Additionally, the control of Li+TFSI^−^ solubility in an IG on a BCP PC facilitates the temperature-interactive SC change of the PC [47]. Park et al. developed a temperature-interactive BCP SC display with a poly(N-isopropyl acrylamide) (PNIPAM) blended with Li+TFSI^−^ on a BCP PC. A blended layer of PNIPAM/Li+TFSI^−^ showed its characteristic phase separation behavior, wherein two materials were phase-segregated below a certain transition temperature while they were completely mixed above the transition temperature. The as-cast blended film of PNIPAM/Li+TFSI^−^ was opaque due to the macro-phase separation of PNIPAM and Li+TFSI^−^ with Li+TFSI^−^ rich and poor domains at room temperature. When temperature approached the transition temperature of a PNIPAM/Li+TFSI^−^ layer of approximately 40°C, the film in a gel state became transparent into a sol state. In the sol state, the solubility of Li+TFSI^−^ in PNIPAM was substantially enhanced. When the temperature was increased from 20°C to approximately 40°C with a bilayer of PNIPAM/Li+TFSI^−^ and BCP PC, water molecules started to evaporate from Li+TFSI^−^ dissolved in the BCP PC; this resulted in a blue-shift in its SC. When the temperature was above the transition temperature of approximately 40°C, the PNIPAM/Li+TFSI bilayer became homogeneous (high solubility) and the Li+TFSI^−^ ions migrated from the QP2VP domains (low solubility) to the PNIPAM layer; this resulted in a more significant blue-shift in SC. The temperature-interactive SC variation was reversible and allowed for reliable and repetitive full-color SC modulation with temperature.

**Figure 9.**
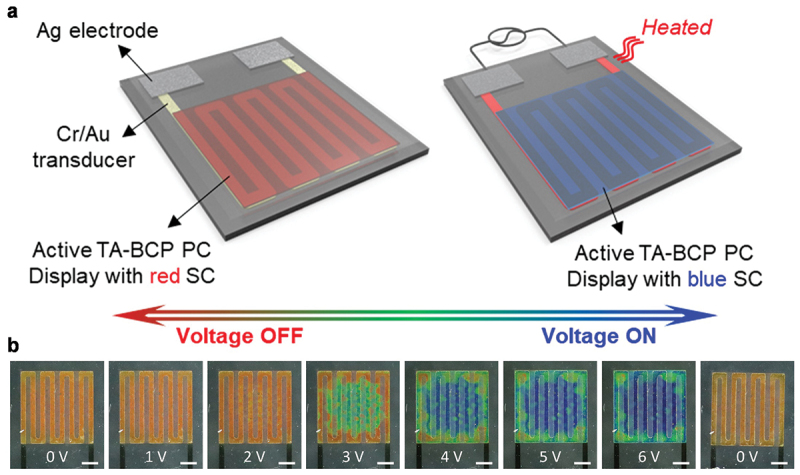
(a) Schematic of the temperature-interactive BCP SC display consisting of a patterned Cr/Au heater, BCP PC, and PNIPAM/Li+TFSI^−^ IG. (b) Photographs of the temperature-interactive BCP SC display with the applied voltage increasing from 0 to 6 V. Reproduced with permission [47]. Copyright 2022, Wiley-VCH.

A temperature-interactive BCP SC display was fabricated using the transducer (heater) enabling electrical-to-thermal energy conversion. Initially, a temperature-interactive BCP SC was placed on a parallel Cr/Au electrical heater with a lithographically defined patterned electrode (Figure 9(a)). When applying a voltage to the Cr/Au electrodes, resistive Joule heating occurred, and the heat from Cr/Au was subsequently transferred to the BCP SC display; this resulted in a blue-shift in the SC. Temperature-interactive SCs obtained in the full-visible range were red, green, and blue as the voltage was increased from 0 to 6 V (Figure 9(b)). When no voltage was applied, the display turned to the initial red SC. The SC changes reliably occurred with multiple voltage sweeps of 0 to 6 V using over 100 cycles with no noticeable alteration in the surface topology. This study also successfully demonstrated the suitability of the display for visualizing body temperature and localized heat in micro-electrical circuitry.

### 3.3. Mechanical force-interactive BCP display

**Figure 10.**
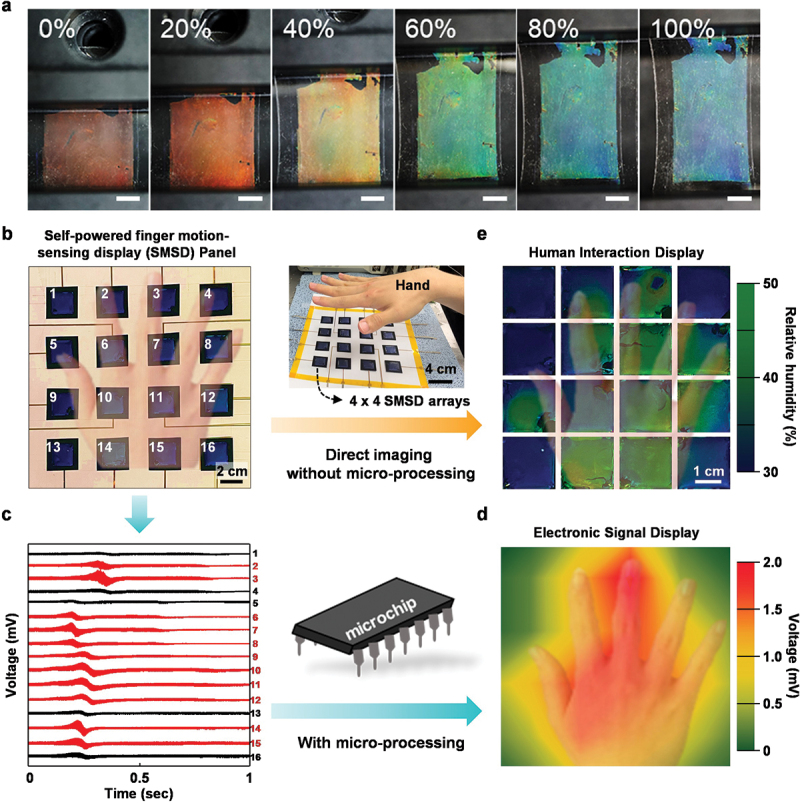
(a) Series of photographs of mechanical force-interactive BCP SC display as a function of strain. Reproduced with permission [48], copyright 2018, Springer Nature. (b) Photograph of the pixelated 4 × 4 arrays of individual SMBD units on a flexible substrate (20 × 20 cm^2^). (c) the output voltages of the 16 SMBD units when the palm is getting close to the surface. (d) 2-dimensional (2D) contour mapping based on the output voltage from the 16 SMBD units by the palm motion in (c). (e) Photographs of BCP SC of the SMBD visualized with a non-contact mode palm motion. Reproduced with permission [13]. Copyright 2022, Elsevier.

A mechanical force-interactive BCP SC display was developed by embedding Li+TFSI^−^-doped BCP in mechanically flexible and stretchable elastomer of polydimethylsiloxane (PDMS); the SC of the BCP PC was responsive to either strain or pressure with high cycle reliability [48]. The tensile strain applied along the x- or y- axis resulted in compression along the z-axis, reducing the thickness of the BCP PC. When the strain increased from 0 to 100%, the SC of the BCP PC changed from red to blue owing to the compression of the swollen Li+TFSI^−^-doped QP2VP layers; this allowed the visualization of the mechanical strain in SC (Figure 10(a)). It should be noted that the mechanical deformation behavior of the BCP PC consisted of alternating glassy PS and soft QP2VP layers. The unique architecture of the display with a BCP PC laminated and embedded with the elastomeric PDMS structure, the plasticization of QP2VP layers by the Li+TFSI^−^ ions, and the mechanical cracks on the glassy PS layers were responsible for the excellent mechanical force-interactive SC of the display.

The mechanical force-interactive BCP SC display was suitable for wearable applications; it could detect and visualize the motion of the human finger, elbow, and knee. To obtain an electrical signal change under strain and pressure, ion-conductive hydrogels were employed underneath the PDMS. When the mechanical force-interactive BCP SC display was subjected to mechanical deformation, the change in the capacitance and SC resulted from the alteration in the dimension of the display; this was quantitatively measured and visualized. For example, the strain due to finger-bending was detected via the capacitance and an SC change from red to green. Furthermore, this provided a convenient way to obtain positional information on the strained areas without the pixelated arrays. When a push – pull gauge pressurized the four different positions with the same pressure, the capacitance change at all four positions was very similar. The optical information on BCP SC changes enabled positional recognition. Both the capacitance and SC information were achieved by gently touching four rubber stamps with similar areas and different shapes (a topographic clover, tree, star, and stick) onto the BCP SC display. The four shapes were visualized in the SC, and no significant differences were observed in the capacitance due to the constant pressure on the samples. SC-based position and shape recognition were unique properties of the mechanical force-interactive BCP SC display, compared to conventional strain sensors based on electrical signals, such as resistance, capacitance, and impedance.

### 3.4. Self-powered motion-interactive BCP display

A self-powered motion-interactive BCP SC display (SMBD) was developed, where Li+TFSI^−^-doped IHN-BCP SC was fabricated on the IG electrode [13]. The SMBD was based on motion-interactive triboelectrification; the SC of the IHN-BCP PC allowed for optical recognition of various gestures, such as vertical and sliding motions of the fingers. For Li+TFSI^−^-doped IHN in BCP PC, an ionic solution containing IHN monomers and Li+TFSI^−^ was spread onto the BCP PC surface followed by UV curing; this produced humidity-interactive BPC PC displays, as discussed previously.

The position of a finger with natural humidity was observed with the humidity-interactive SC. Finger motion was quantified with the triboelectrification of an SMBD. The SC was red-shifted when the finger approached the surface of an SMBD due to the increase in humidity. When the finger moved toward the SMBD with displacements of 2, 1, and 0.5 cm, the SCs of the SMBD turned blue, green, and red, respectively. Approximately 70, 50, and 30% RH were measured with a finger position of 0.5, 1, and 2 cm from the SMBD, respectively. The triboelectric output voltage of the SMBD increased as the displacement was closer because electrostatic induction was enhanced when a charged object moved closer to another object. The triboelectric output voltages of vertical and sliding motion were systematically examined as a function of the distance between the SMBD and finger. Furthermore, the triboelectric signals and BCP SCs obtained from 4 × 4 arrays of the SMBDs enabled the detection and visualization of various finger and hand motions (Figure 10(b)). The pixelated SMBD arrays were operated in a non-contact mode, wherein various finger and hand motions were characterized by the triboelectric output voltage of the 16 individual devices (Figure 10(c)). The position of finger motion was identified in the 16 devices from their output signals. Subsequently, the output voltages were converted and transferred using a microprocessor; the 2D contour map based on the output voltage was visualized (Figure 10(d)). Also, a humidity-interactive BCP in the SMBD directly visualized hand motion depending on the humidity of the finger without a microprocessor, as shown in Figure 10(e).

The SMBD was connected to different external loads for harvesting the self-powered operation energy. When the display was periodically tapped on a counterpart film with a cycled compressive force of 1 N at a frequency of 2 Hz, electric power was generated and subsequently stored in a capacitor. The voltage of the capacitor gradually increased with the tapping events. When charging at 1 µF for 30 sec was conducted, the capacitor charged the voltage to approximately 0.43 V at the RH of 80%. The SMBD exhibited excellent charging and discharging cycles for 1 µF commercial capacitor. The capacitor was charged to 0.26 V in 25 sec; when the force was removed, the capacitor discharged the stored potential to approximately 0.02 V.

## 4. Perspective

In addition to the significant progress in developing programmable and stimuli-interactive SC displays based on 1D PCs of self-assembled BCPs, several issues should be considered for further enhancing the performance of the displays. For the programmable displays, key operation properties, including the high reflectance of visible SC, fast SC switching speed, reliable write/erase cycle endurance, high SC resolution, and scalability of the displays with pixel arrays and circuit integration, should be assured. Along with the properties mentioned previously for programmable displays, stimuli-interactive displays should also have the properties required for a sensor, including high sensitivity, fast response time, and sensing reliability.

A novel design of materials, structures, and processes should be developed to ensure these key properties. Understanding the diffusion mechanism of swelling agents into a 1D BCP PC consisting of alternating non-swelling and swelling layers is of great importance. Recent studies have shown that the characteristic screw dislocations in a 1D PC were responsible for the fast infusion of a swelling agent; this allowed the fast SC alteration of a BCP PC [51]. Further enhancement of SC-switching speed can be made by properly controlling the position and areal density of these screw dislocations. Another way to facilitate SC switching in a BCP SC display is to develop topological micropatterns of the BCP PC films; a swelling agent is laterally diffused into the micropatterns through the side walls of the patterns [50]. Studies on the SC alteration of micro/nanopatterned BCP PCs with various shapes, symmetries, and dimensions should be performed. To enhance the reflectance of a BCP SC, the selective doping and hybridization of nanomaterials into one of the two lamellae have been effective in increasing the dielectric constant contrast between the BCP SCs with nanomaterials and others, such as various high-k dielectric nanomaterials, semi-conducting quantum dots, and metal nanoparticles [52,53]. Recently, organic – inorganic hybrid perovskites have been successfully incorporated into BCP nanostructures. Nanomaterials embedded into BCP PCs interact with diverse stimuli, such as the photoluminescence of semiconducting quantum dots and hybrid perovskites and the magnetic susceptibility of iron oxide nanoparticles [54–57]. These stimuli-interactive properties of the nanomaterials in BCP PCs allow for the development of novel interactive SC displays of BCP PCs.

Recently, photonic crystals based on self-assembled bottle-brushed BCPs have been synthesized, producing visible range SC [19–24]. The bottle-brushed BCPs allowed for a facile control of the periodicity of a PC, compared with that of a di-BCP PC. BCP SC displays could also be developed with 2D and 3D PCs rather than 1D PCs with alternating lamellae [20,23,24]. Due to the faster and easier swelling of BCP domains with infusing swelling agents, a faster SC switching of 2D and 3D PCs is expected over 1D PCs. A 3D PC with a bicontinuous gyroid nanostructure has been demonstrated with facile SC variation and a swelling solvent [58]. Solution-processible BCP PC can be synergistically combined with conventional display technologies, such as light emitting diodes, holograms, and electrochromic technologies; this combination can produce unprecedented SC displays.

## 5. Summary

The development of SCs with self-assembled 1D BCP PC has become a research focus due to the facile and diverse nano-architectonics relying on the volume ratio of blocks and excellent capability to change their nano-architectonics in response to various external stimuli. Thus, we provided a comprehensive overview of the recent advances in actively programmable and stimuli-interactive BCP SC displays. Initially, this review focused on the development of programmable BCP SCs that stored various information, such as color, pH, photo-induced crosslinking, chemical crosslinking, and E-field. Further, we addressed the stimuli-interactive BCP SCs that responded reversibly to external stimuli, including temperature, mechanical force, and humidity. Finally, based on previously reported literature, the current challenges and next-generation of BCP SC displays are presented from the following perspectives: 1) understanding the diffusion mechanism of swelling agents and the topological micropatterns for improving the BCP SC-switching kinetics, 2) hybridization of nanomaterials to enhance reflectance and develop new stimuli-interactive properties, and 3) design of new materials and nano-architectonics with 2D and 3D BCP PCs rather than 1D PCs with alternating lamellae.
